# The Associations of Emotion Coping Appraisal With Both the Cue–Outcome Contingency and Perceived Verbal Abuse Exposure

**DOI:** 10.3389/fpsyt.2019.00250

**Published:** 2019-04-16

**Authors:** Dong Woo Shin, Taekeun Yoon, Bumseok Jeong

**Affiliations:** ^1^Graduate School of Medical Science and Engineering, Korea Advanced Institute of Science and Technology (KAIST), Daejeon, South Korea; ^2^KAIST Institute for Health Science and Technology, KAIST, Daejeon, South Korea; ^3^KAIST Clinic Pappalardo Center, KAIST, Daejeon, South Korea

**Keywords:** cognitive appraisal, resilience, hierarchical Gaussian filter, verbal abuse, Bayesian learning, emotion coping appraisal

## Abstract

Previous studies have reported an association between verbal abuse in early childhood and structural and functional alterations in the young adult brain, supporting the existence of critical periods in human brain development. In addition, exposure to verbal abuse in early childhood is strongly correlated with lifetime psychiatric illness. Resilience is defined as the ability to avoid the negative psychological, biological, and social consequences of stress that impair psychological and physical homeostasis and is used to cope with these psychiatric diseases. We attempted to explain the mediatable present function of resilience and its associations with several psychiatric disorders, with verbal abuse exposure in early childhood and with the present value of the readily measurable and conceptually connected generative Bayesian model parameter. Thirty-six subjects performed a cross-modal associative learning task requiring them to learn the predictive strength of auditory cues and predict a subsequent visual stimulus. The probability of the association changed across each trial block. Subjects’ responses were modeled as a hierarchical Bayesian belief-updating process using a hierarchical Gaussian filter (HGF) with three levels, a Sutton K1 model, and a Rescorla–Wagner model. Subjects completed the Korean version of the Verbal Abuse Questionnaire (VAQ) for segmented periods (aged 0 to 6, 7 to 12, and 13 to 18 years), and their positive self-appraisal was estimated using the Resilience Appraisal Scale (RAS). Random-effects Bayesian model selection identified HGF as the best model. Of the VAQ values for specific periods, only preschool VAQ scores were negatively correlated with RAS scores. The tonic volatility parameter, ω_2_, of HGF showed a negative relationship with RAS emotion coping scores. The linear regression model explained 18.3% of the variance of emotion coping appraisal with ω_2_ and preschool VAQ scores. Based on the results obtained from young adults, decrease in emotion coping appraisal can be explained by an increase in the number of experiences of verbal abuse in early childhood and the increased tendency to update beliefs about the cue–outcome associative probability in a volatile environment.

## Introduction

The core modulators of stressful experiences are predictability and controllability (1), which are associated with the uncertainty of inputs from the external world. Stress-adaptive behaviors are formed through model updates that predict and estimate uncertainty and precision-weighted prediction error. Therefore, updating the model appropriately in an uncertain environment is critical for preventing stress-induced maladaptive processes (2), which are potentially linked to stress-related disorders (3).

Many studies examining computational models of this adaptive behavior for input derived from an uncertain environment have employed physically painful feedback (4, 5) social stress (4), and financial reward (2). In real life, however, we mostly assess uncertainty in decision-making without painful feedback, such as electric shock, in contrast to the experimental environment. Similarly, neither a monetary reward nor social stress is a factor contributing to every decision. According to recent studies, individuals track separable forms of uncertainty sufficiently during tone–picture association learning within a nonstressful context because the uncertain environment is sufficiently stressful for the subjects (6, 7).

The dynamic nature of processing uncertainty in an individual varies as the organism learns about and interacts with its environment (8). Individual differences in the estimation of uncertainty might provide insights into individual resilience in a stressful environment (9). According to the cognitive appraisal theory, the quality of the emotion felt by individuals in an unforeseen environment is generated by an individual explanation of its source, feelings about the explanation, and the interpretation of the experienced arousal (10). Therefore, in the context of the emotions experienced by subjects in relation to uncertainty, individual variation in parameters estimated with computational modeling may be associated with individual differences in the adaptive process.

Resilience is a necessary skill to maintain an individual’s psychological and physical condition in response to external stress (9). Moreover, resilience varies in humans, representing individual differences in an adaptive and active process in an uncertain or stressful situation (11, 12). Resilience may be an individual trait in that non-uniform neuroendocrine findings related to resilience have been found, and the relationship between individual resilience and psychiatric disorders such as anxiety and depression has been confirmed (9). However, little research has been performed to evaluate the relationship between the aforementioned uncertainty and individual resilience, although persons show individualized responses to uncertainty during tone–picture association learning in a nonstressful context (6, 7). We argue that this association needs to be studied because individual differences in model updating under stress in an uncertain situation are related to the meaning of individual resilience.

A growing body of research has begun documenting how early life stress, including emotional maltreatment, can increase vulnerability to stress-related disorders along with anatomical and functional abnormalities in the brain. The results of early stress exposure may help individuals cope with stress or increase their sensitivity to the negative effects of adult stress (13). However, excessive stress in early life exerts negative effects on changes in hippocampal structure in both animals (13, 14) and humans (15, 16). Aberrant changes in white matter structural connectivity in young adults who have experienced childhood maltreatment but do not suffer from mental disorders have also been reported in cortico-limbic or cortico-cortical connections, such as the uncinate fasciculus (17), the cingulum bundle (18), and the superior (18) and inferior (19) longitudinal fasciculus. Moreover, aberrant functional activity and connectivity have been reported in the medial frontal-amygdala circuit during the processing of contemptuous facial expressions (20), and aberrations have also been observed in ventrolateral prefrontal activity and its connectivity with the hippocampus during exposure to swear words in healthy adolescents who have been exposed to verbal abuse (21). An increase in corticotropin-releasing hormone levels in the cerebrospinal fluid of adults with a history of childhood abuse was reported (22), although this increase was not always associated with diseases such as posttraumatic stress disorder or major depressive disorder (23, 24).

Childhood maltreatment by caregivers is particularly considered an abuse experience that is marked by unpredictability and uncontrollability because children are not independent from their caregivers. Children who have experienced childhood abuse tend to be unable to cope appropriately with the stressful situations and tend to take a passive stance about daily stress due to failure to meet earlier developmental milestones (25, 26). These tendencies may lead to psychiatric problems. According to one study, parental verbal abuse at age 5 was the greatest predictor of suicidal ideation in young men with a history of childhood maltreatment (27). In addition, the odds of suicidal ideation were 2.5 times higher in young adults who had experienced sexual abuse in the preschool period than those who had had similar experiences in adolescence (28).

In particular, verbal abuse is more potently associated with scores on the psychological symptom scale than witnessing domestic violence or physical abuse (29). Additionally, verbal abuse potentially exerts a negative impact on the development of specific brain regions and increases the likelihood of developing a psychiatric disease (30). Since verbal abuse is a form of emotional maltreatment and a potent precursor of dissociation, this form of abuse has a stronger association with psychiatric sequelae than other forms of abuse (31, 32). Emotion coping appraisal, a component of resilience, is an important scale with which to evaluate individuals who have been verbally abused. Individuals use stimulus evaluation checks, such as a capacity for control and prediction error, to interpret external emotional stimuli, and consequently, their emotional state is adjusted by the function of emotion coping appraisal according to the cognitive theory of emotion (33, 34).

**Figure 1:**
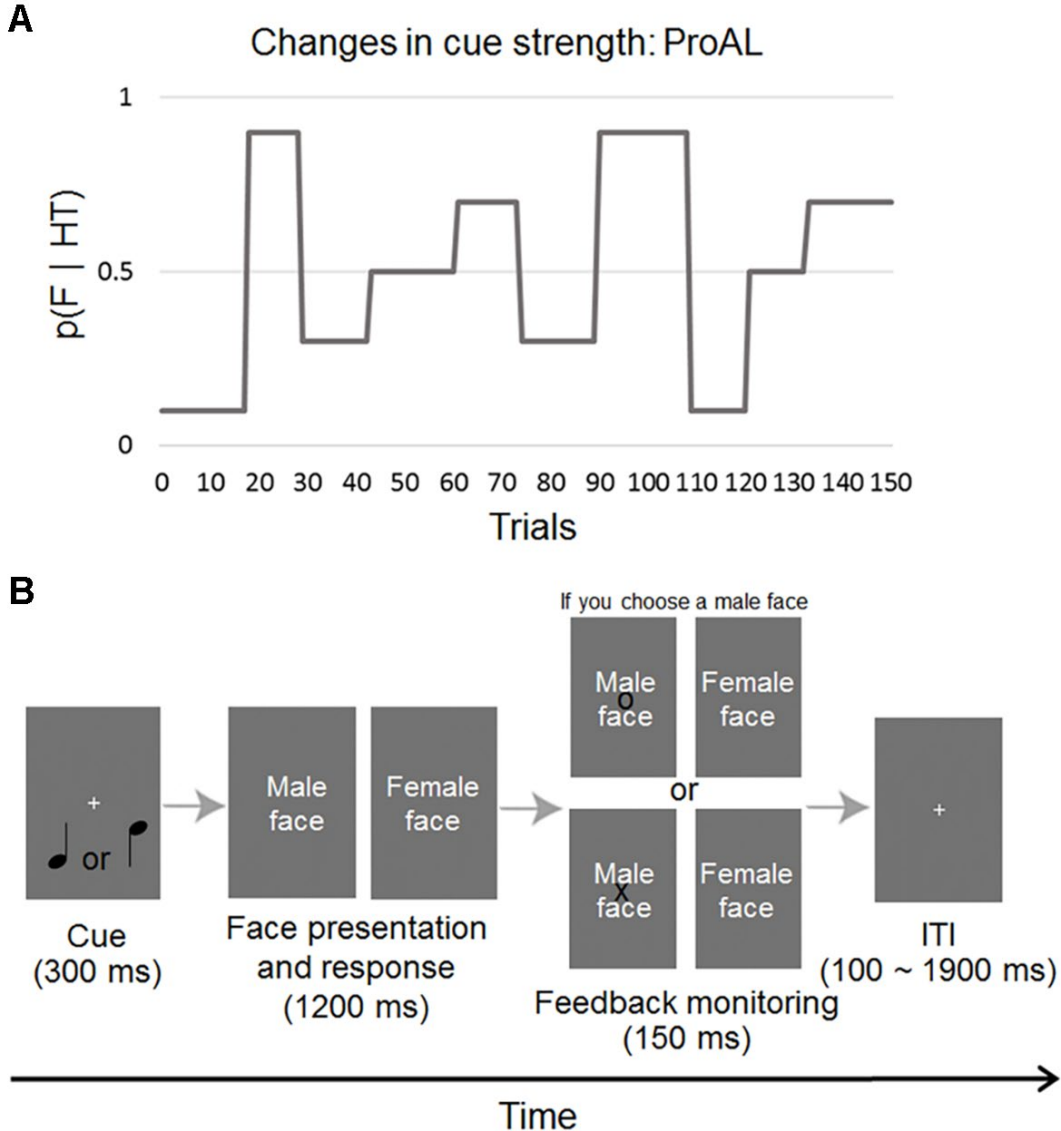
Structure of the ProAL task. **(A)** The probability that a female (F) image is presented after a high tone (HT) is represented by *p* (F|HT), and the change in *p* (F|HT) over time is displayed. **(B)** Subjects performed a cross-modal ProAL task. This task consists of cue, face presentation and response, monitor feedback monitoring, and an ITI. This task allows the subject to learn the predictive strength of auditory cues and predict a subsequent visual stimulus. ITI, intertrial interval.

**Figure 2:**
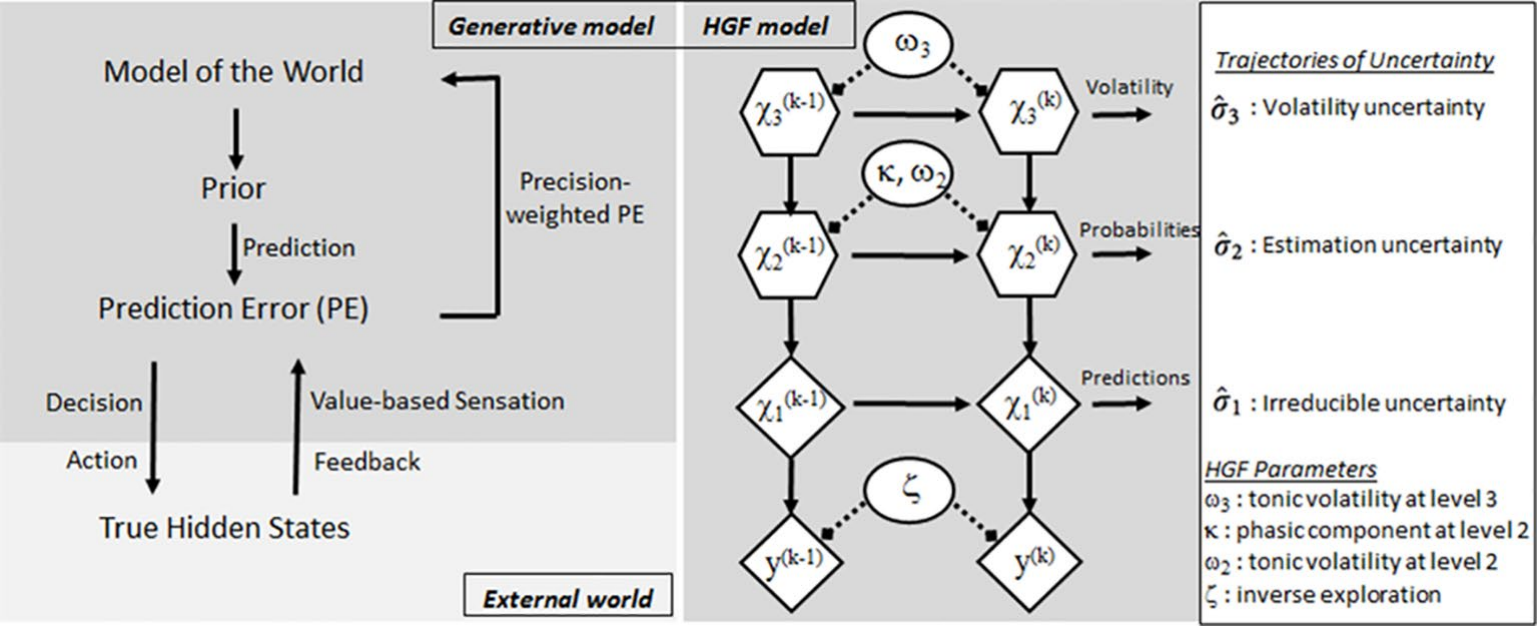
Three-level hierarchical Gaussian filter (HGF).

The probabilistic associative learning (ProAL) task enables learning and decision-making in situations where the cue–outcome contingency (cue–outcome associative probability) changes stochastically (see **Figure 1A**). Subjects feel that the cue–outcome contingency is uncertain when they perform the ProAL task, and this situation is the volatile environment. We tried to quantify individual differences in learning parameters under these conditions by ensuring that subjects feel uncertainty in a volatile environment. Several computational studies of decision-making processes have shown that subjects tune their learning rate about cue–outcome contingencies in response to a volatile environment (35–37). However, these models are not suitable for describing learning in a volatile environment (38). Recent hierarchical models have quantified individual differences in this volatile learning (5, 6, 39) and a hierarchical Gaussian filter (HGF, see **Figure 2**) with three hierarchical probability distributions is representative of various Bayesian inference models (6, 39, 40) (see **Figure 2**). Research has already been conducted to identify learning-based indicators of individuals with psychiatric disorders and to identify their characteristics (38).

**Figure 3:**
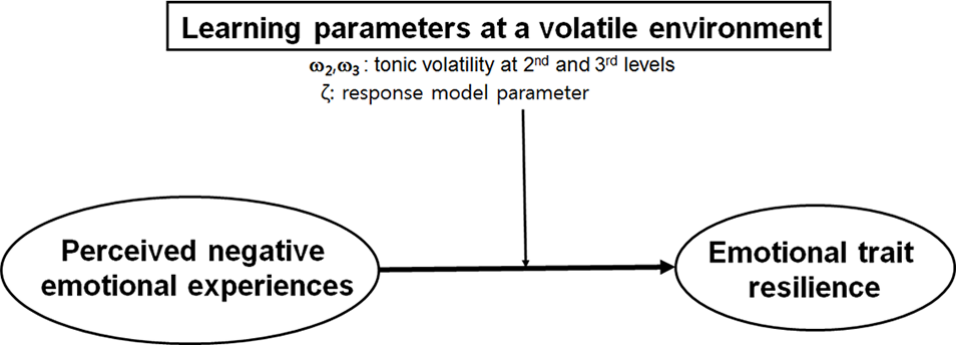
Our hypothetical scheme.

Based on these considerations, we hypothesized that emotion coping appraisal is correlated with HGF parameters derived from the uncertain environment and verbal abuse exposure, particularly during early childhood. If our hypothesis is validated, the “behavior” of emotion coping appraisal would be explained by the “present” learning tendency in a volatile environment and perceived “past” verbal abuse exposure in early childhood (see **Figure 3**).

## Materials and Methods

### Subjects

Thirty-six healthy subjects (mean age ± standard deviation = 23.4 ± 4.1 years, M/F = 23:13) participated in our experiment. The subjects were interviewed by skilled psychiatrists using the Korean version of the Diagnostic Interview for Genetic Studies (DIGS-K version 2.0) (41). The subjects did not have psychiatric or neurological illnesses, and their visual acuity or corrected vision was normal (inclusion criteria). No subjects were excluded based on the screen administered by skilled psychiatrists. Subjects were recruited from the University of Korea Advanced Institute of Science and Technology (KAIST) and provided written and informed consent. This research was approved by KAIST Institutional Review Boards (approval number: KH2013-23) and was conducted in accordance with the Declaration of Helsinki.

### Behavioral Task: ProAL

The subjects performed an associative learning task in which the degree of association between two types stimuli, consisting of two auditory stimuli (low or high tones) and two visual stimuli (male and female neutral faces), changes over time. We used the human faces as stimuli in the task to study the relationship between the emotion coping ability and the learning parameters of the task. Subjects were required to constantly track the relationship over time, resulting in fluctuations in the levels of various forms of uncertainty. The probabilities governing each block varied between 90/10, 70/30, and 50/50 to examine the dynamic change in uncertainty, as described in a previous study (see **Figure 1A**) (5). During feedback, subjects received information in the form of an “O” or “X” mark on the image of the face according to the correct answer (see **Figure 1B**). Each session of 150 trials was divided into 10 blocks of different stimulus–outcome probabilities of lengths ranging from 11 to 17 trials. The subjects performed a sufficient number of practice sessions, and we used the subsequent behavioral data obtained in the MRI scanner for analysis. A functional neuroimaging study was performed on the same subjects and the results will be published elsewhere.

### Behavioral Modeling

We use three models, Rescorla–Wagner (RW), Sutton K1 (SK-­1), and HGF, which are implemented in HGF module version 5.1 of Translational Neuromodeling Unit’s Algorithms for Psychiatry-Advancing Science toolbox (https://www.tnu.ethz.ch/en/software/tapas.html), to model learning in the ProAL task. The HGF used here is the three-level generative model based on Bayesian inference. When a new stimulus is presented, learning at three levels of the uncertainty hierarchy occurs simultaneously with two free perceptual model parameters, ω_3_ at the third level and ω_2_ at the second level, and a response model parameter ζ. The first level of HGF (*x*_1_) is the process of recognizing external stimuli and addresses uncertainty about outcomes (e.g., female or male image). The second level of HGF (*x*_2_) quantifies cue–outcome associative probability (e.g., the probability of female–high tone match) and addresses uncertainty about probabilities (cue–outcome contingencies). The third level of HGF (*x*_3_) represents the volatility of the probabilities (the degree of change of the second level). In other words, the third level addresses uncertainty about the environmental change. For each trial, predictions of each level occur for the next trial and are represented by a Gaussian distribution. The mean and variance of this distribution at each level are called ${\hat{\mu }}_{i}$ and ${\hat{\sigma }}_{i}$, respectively. ${\hat{\sigma }}_{i}$ quantifies the uncertainty of the estimate for each three level. In each stage, beliefs are updated through prediction errors. In our HGF model, the stimulus category *x*_1_ at time *k* is denoted by $x_{1}^{\left(k\right)}\in \{0,1\}$.

*s*(·) is a sigmoid function:

$$s(x)\overset{def}{=}\frac{1}{1+\exp(-x)}$$

The three-level hierarchy of the HGF model is described using the following equations:

$$x_{1}^{\left(k\right)}∼Bernoulli\left(s\left(x_{2}^{\left(k\right)}\right)\right)$$

$$x_{2}^{\left(k\right)}∼N\left(x_{2}^{\left(k-1\right)},\exp\left(\kappa x_{3}^{k}+\omega_{2}\right)\right)$$

$$x_{3}^{\left(k\right)}∼N\left(x_{3}^{\left(k-1\right)},\exp(\omega_{3})\right)$$

By definition, ω_2_ and ω_3_ represent the characteristics of subjects in the learning process for a particular situation. The value of ω represents a step size of a Gaussian random walk, and a higher value of ω is more likely to represent an unstable cue–outcome association (39). κ, the phasic component of the second-level probability distribution, was fixed. Finally, the response model was modeled using a unit square sigmoid function such that

$$p(y^{\left(k\right)}=1)=\frac{{\left({\hat{\mu }}_{1}^{\left(k\right)}\right)}^{\zeta }}{{\left({\hat{\mu }}_{1}^{\left(k\right)}\right)}^{\zeta }+{\left(1-{\hat{\mu }}_{1}^{\left(k\right)}\right)}^{\zeta }}$$

where ζ is interpreted as inverse decision noise. We described the means and variance of Gaussian priors used in HGF parameter estimation in **Supplementary Table 1**.

## Model validation: random-effect Bayesian model selection

The HGF was compared with SK-1 and the RW model, which is known to best explain probability-based learning among classical learning theories, to confirm that the behavioral tasks used in the experiments are appropriate to be analyzed with HGF assuming the Bayesian brain. For this comparison of the models, the random-effect Bayesian model selection (RFX-BMS) embedded in the VBA toolbox (mbb-team.github.io/VBA-toolbox/) was used (42, 43). We used log-model evidence approximated by the negative variational free energy under the Laplace assumption to compare the models.

### Clinical Variables

The Resilience Appraisal Scale (RAS), the Korean version of Verbal Abuse Questionnaire (VAQ) to measure the perceived verbal abuse experiences, and Spielberger’s State–Trait Anxiety Inventory (STAI-S/-T) were administered to subjects (see **Table 1**).

**Table 1:** Demographic and clinical characteristics of the subjects.

	Mean (SD)	Median	Min/max	Skewness	Kurtosis
Age	23.42 (4.10)	22.38	18.32/34.37	0.84…	−0.14
Sex (male/female)	23:13	—	—	—	—
VAQ-preschool	5.50 (10.39)	3.00	0/58	3.76 …	15.50
VAQ (T)-preschool	1.31 (0.98)	1.47	0/4.14	0.42 …	0.27….
VAQ-childhood	13.86 (16.80)	9.00	0.00/82.00	6.39 …	2.80….
VAQ-adolescent	14.36 (15.04)	10.00	0.00/73.00	2.06 …	4.76…
RAS-total	48.86 (5.84)	49.00	37.00/60.00	−0.14 …	−0.67…
RAS-emotion coping	15.44 (2.52)	16.00	10.00/20.00	−0.41 …	−0.50…
RAS-social support	17.06 (2.10)	17.00	13.00/20.00	0.02 …	−1.25…
RAS-situation coping	16.36 (2.10)	16.00	10.00/20.00	−0.67 …	1.16…
STAI-S	35.83 (9.19)	36.50	20.00/60.00	0.47 …	0.08…
STAI-T	36.00 (9.63)	36.00	20.00/60.00	0.57 …	−0.06…

SD, standard deviation; VAQ, Verbal Abuse Questionnaire; (T), Tukey’s ladder of powers transformation; RAS, Resilience Appraisal Scale; STAI-S, Spielberger’s state–trait anxiety inventory—state; STAI-T, Spielberger’s state–trait anxiety inventory—trait.

#### RAS

The RAS (44) is a 12-item scale consisting of three 4-item subscales estimating positive self-appraisals. These subscales focus on self-appraisals of the individual’s perceived ability to cope with emotions, solve difficult situational problems, and obtain social support. An example item for assessing emotion coping appraisals is “I can handle my emotions,” one for situation coping appraisals is “When faced with a problem, I can usually find a solution,” and one for social support appraisals is “If I were in trouble, I know of others who would be able to help me.” Responses were rated on a five-point scale from “strongly disagree” to “strongly agree.” Alpha reliabilities of RAS-total (α = 0.88), RAS-emotion coping (α = 0.92), RAS-situation coping (α = 0.92), and RAS-social support (α = 0.93) scores were significant (44). This scale has been used in young and older adults (45).

#### Verbal Abuse Questionnaire

Lifetime experiences of perceived verbal abuse among preschoolers (aged 0–6 years), children (aged 7–12 years), and adolescents (aged 13–18 years) were measured using the VAQ that has been validated for the Korean college population (29, 46). The VAQ is composed of 15 items covering scolding, yelling, swearing, blaming, insulting, threatening, demeaning, ridiculing, criticizing, and belittling; the perceived severity was reported using a nine-point Likert scale (from 0 = “not at all” to 8 = “every day”) (46). Subjects reported perceived parental verbal abuse exposure at the present time. Among the clinical variables, VAQ-preschool was transformed using Tukey’s “ladder of powers” method (47) because of high kurtosis and skewness.

#### Spielberger’s State–Trait Anxiety Inventory

The Korean version (48) of STAI (49), a validated questionnaire, consisted of 20 questions. The STAI-S (not at all, somewhat, moderately so, and very much so) and STAI-T (almost never, sometimes, often, and almost always) consist of a four-point scale. STAI-S indicates state anxiety and STAI-T represents trait anxiety. The scores ranged from 20 to 80 points, with higher scores indicating anxiety. Because anxiety is associated with emotion coping (50), we have measured it to confirm the indirect association with other factors using a mixed graphical model analysis.

### Statistical Analyses

#### Simple Correlation Analysis

Pearson correlation analyses were performed among HGF parameters (ω_2_, ω_3_, and ζ), RAS (total, emotion coping, situation coping, and social support appraisals), VAQ (preschool, childhood, and adolescent), STAI-S, STAI-T, and age.

#### Linear Regression Analyses to Predict Individual’s Positive Coping Appraisals

First, we tested multicollinearity with the variance inflation factor (VIF) of a predictor variable in the following model consisting of all VAQ scores and HGF parameters:

RAS ∼ VAQ-preschool (T) + VAQ-childhood + VAQ-adolescent + ω_2_ + ω_3_+ ζ

[(T): Tukey’s ladder of powers transformation]

The multicollinearity test showed that high VIF values for VAQ-childhood (7.04) and VAQ-adolescent (7.01), and models for predicting RAS-total [*F*(6, 29) = 1.458, *p* = 0.2273] and subscale scores [RAS-emotion coping: *F*(6, 29) = 2.031, *p* = 0.0935, RAS-social support: *F*(6, 29) = 0.8918, *p* = 0.5138, RAS-situation coping: *F*(6, 29) = 0.9834, *p* = 0.4544] were not significant. If the VIF of a predictor variable was 7.04 (√7.04 = 2.65), then the standard error of the coefficient of that predictor variable was 2.65 times larger than it would be if that predictor variable was not correlated with the other predictor variables. Based on results from correlation analyses and from the multicollinearity test for the models described above, we produced four different models including preschool VAQ scores (VAQ-preschool) and ω_2_, as shown below.

Simple model: RAS ∼ VAQ-preschool (T) + ω_2_

Full model: RAS ∼ VAQ-preschool (T) + ω_2_ + VAQ-preschool (T) × ω_2_

Partial model with ω_2_: RAS ∼ ω_2_ + VAQ-preschool (T) × ω_2_

Partial model with VAQ-preschool: RAS ∼ VAQ-preschool (T) + VAQ-preschool (T) × ω_2_

[VAQ-preschool (T) × ω_2_: interaction of VAQ-preschool (T) and ω_2_, (T): Tukey’s ladder of powers transformation]

With the exception of the “Full model” that showed multicollinearity (see **Supplementary Table 2**), the remaining three models were tested to predict RAS-total and subscale scores. Anxiety scores (STAI-T/-S) are not included as predictors in regression models because the tendency towards anxiety is well known to be associated with cognitive coping (50, 51) and because the inclusion of anxiety scores as predictors increases model complexity. The best model was selected when its adjusted *R* ^2^ had the highest value. Analysis of variance (ANOVA) was used to compare regression models. Using leave-one-out cross-validation (LOOCV), a prediction score, *Q* ^2^, was also calculated. All statistical analyses were performed in R (version 3.5.0) (52).

#### Mixed Graphical Model Analysis

The role of gender should be considered in reappraisals, such as emotion regulation (53). State or trait anxiety is also related to reappraisal (51) and decision-making (54). Thus, using a mixed graphical model (“mgm” package of version 1.2-2 in R 3.5.0) for data including variables with a non-Gaussian distribution, we determined conditionally independent relationships among STAI-S or -T, gender, and predictors of the best model chosen in linear regression analyses. Here, we assume that at most, pairwise interactions exist in the true graph. The algorithm including an L1-penalty was used to obtain a sparse estimation, and the Extended Bayesian Information Criterion was applied to select the optimal regularization parameter lambda.

## Results

### Performance on the ProAL Task

Among 150 trials, the accuracy and number of missing trials were 54.98 ± 4.98% [mean ± standard deviation, 45.95 (min)–64.67 (max) %] and 0.83 ± 1.65 [mean ± standard deviation, 0 (min)–9 (max)], respectively.

### Behavioral Modeling

Three models, RW, SK-1, and three-level HGF, were compared in a random-effect model (42). In RFX-BMS [Bayesian omnibus risk: *p* (H0|*y*) ≥ 0.025], the best model was the three-level HGF model (estimated model frequencies: 0.710, protected exceedance probabilities: 0.979; see **Supplementary Table 3** and **Supplementary Figure 1**).

The mean values of three hierarchical learning parameters, ω_2_, ω_3_, and ζ, in the three-level HGF model were −5.46 ± 2.82, −6.06 ± 0.14, and 3.16 ± 1.94 (means ± standard deviations), respectively. The mean log model evidence was −92.59 ± 18.08 (mean ± standard deviation). We conducted a parameter recovery simulation to determine whether the three-level HGF model displayed internal validity. The parameters of the original model were highly correlated with the mean of recovered parameters, indicating that the parameters were reliably estimated (see **Supplementary Figure 2**).

### Correlation Analyses

Only ω_2_ showed a significant relationship with VAQ-childhood (*r* = −0.379, *p* = 0.023), VAQ-adolescent (*r* = −0.448, *p* = 0.006), and RAS-emotion coping scores (*r* = −0.333, *p* = 0.047). ω_3_ and ζ did not show any significant relationship with clinical variables. VAQ-preschool showed a significant relationship with RAS-total (*r* = −0.356, *p* = 0.033) and RAS-emotion coping scores (*r* = −0.361, *p* = 0.030). However, VAQ-childhood and VAQ-adolescent scores were not correlated with RAS scores. STAI-S/-T showed a highly significant relationship with scores for RAS-total (*r* = −0.506/−0.486, *p* = 0.002/0.003) and its subscales [social support (*r* = −0.434/−0.289, *p* = 0.008/0.088), emotion coping (*r* = −0.460/−0.502, *p* = 0.005/0.002), and situation coping (*r* = −0.422/−0.461, *p* = 0.010/0.005)], but not with ω_2_. In summary, ω_2_ and the VAQ-preschool score were negatively correlated with the RAS-emotion coping score, which was negatively correlated with STAI-S and STAI-T scores.

### Regression Analyses for Predicting the Resilience Appraisal Scale–Emotion Coping Score

The model showing the highest adjusted *R* ^2^ value was the “Simple model” for the predicting RAS-emotion coping score (see **Table 2**) and the “Partial model with ω_2_” for predicting the RAS-total score (see **Supplementary Tables 4** and **5**). The best model for predicting the RAS-emotion coping score explains 18.3% of the total variances in RAS-emotion coping scores (see **Table 2**). Cross-validation analyses showed that the “Simple model” predicted 15.6% of RAS-emotion coping scores in a new subject (see **Supplementary Table 6**).

**Table 2:** Multiple linear regression models using RAS-emotion coping as the dependent variable.

	Dependent variable: RAS-emotion coping		
	Simple model	Partial model with VAQ-preschool	Partial model with ω_2_
VAQ-preschool (T) (estimate, 95% CI)	−0.891 (−1.665, −0.116)*	−1.938 (−3.298, −0.578)**	
ω_2_ (estimate, 95% CI)	−0.282 (−0.550, −0.014)*		−0.445 (−0.748, −0.142)**
VAQ-preschool (T) × ω_2_ (estimate, 95% CI)		−0.157 (−0.330, 0.016)	0.119 (0.008, 0.231)*
Constant (estimate, 95% CI)	15.072 (13.102, 17.042)***	16.883 (15.584, 18.182)***	13.851 (12.195, 15.507)***
Adjusted *R* ^2^	0.183	0.158	0.168
Residual standard error (df = 33)	2.281	2.315	2.302
*F* value (df = 2; 33), *p* value	4.923*, 0.0135*	4.286*, 0.0221*	4.528*, 0.0183*

RAS, Resilience Appraisal Scale; VAQ, Verbal Abuse Questionnaire; adjusted R^2^, adjusted squared correlation coefficient; (T), Tukey’s ladder of powers transformation; CI, confidence interval; VAQ-preschool (T) × ω_2_, interaction of VAQ-preschool and ω_2_; Simple model, RAS ∼ VAQ-preschool (T) + ω_2_; Partial model with VAQ-preschool, RAS ∼ VAQ-preschool (T) + VAQ-preschool (T) × ω_2_; Partial model with ω_2_, RAS ∼ ω_2_ + VAQ-preschool (T) × ω_2_; *p < 0.05, **p < 0.01, and ***p < 0.001.

### Mixed Graphical Model (see **Figure 4**)

A mixed graphical model using an undirected graphical model with lasso depicted the associations between emotion coping and ω_2_ and VAQ in preschool and with the subject’s anxiety (STAI-T/-S). The results showing the negative relationships between ω_2_ and VAQ-preschool with RAS-emotion coping were consistent with the results from the “Simple model” in regression analyses. The RAS-emotion coping score was also negatively correlated with trait anxiety (STAI-T). Gender did not have any conditionally independent relationship with other variables in the selected mixed graphical model.

**Figure 4:**
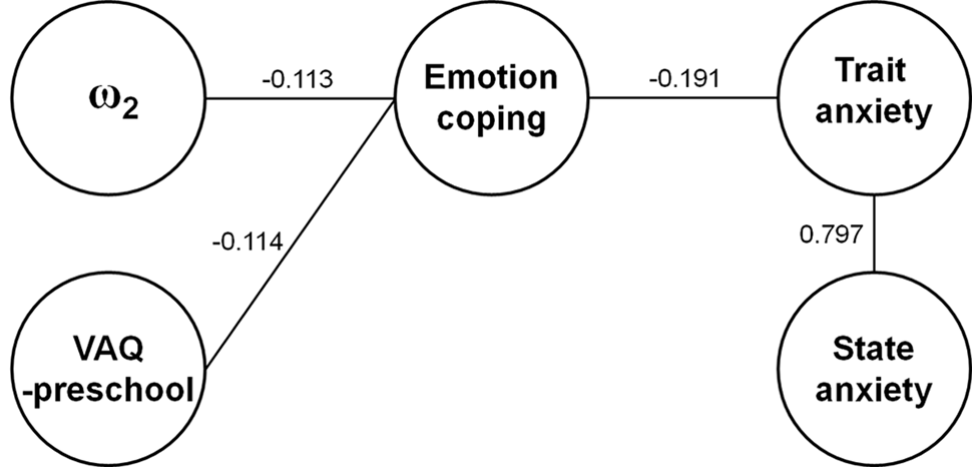
Mixed graphical model of Resilience Appraisal Scale (RAS)-emotion coping. The mixed graphical model described the correlations between the RAS-emotion coping score with ω_2_, Verbal Abuse Questionnaire (VAQ)-preschool, and trait anxiety. The gender factor was removed from the figure as it was not associated with any parameters. The estimates using the mixed graphical model function are displayed above the connecting line.

## Discussion

In this study, using multiple linear regression analysis, we found that decreased emotion coping appraisal of resilience in young adults was explained by an increased tendency to update beliefs about cue–outcome associative probability in a volatile environment and the increase in the parental verbal abuse experienced in early childhood. The total scores of resilience appraisals were also explained in the same manner (see **Supplementary Tables 4** and **7**). In addition, we did not find any effect of gender on the results both with whole subject (**Figure 4**) and in the gender-matched subgroup (see **Supplementary Figure 3**).

Notably, the period in which subjects were exposed to verbal abuse was important in explaining the emotion coping appraisal of the subjects in our study. Only verbal abuse in the preschool period, but not in childhood or adolescence, was significantly associated with RAS-total and RAS-emotion coping scores in our data. Our finding is consistent with studies showing that maltreatment in early childhood is an important factor contributing to social competence associated with neuroendocrine activity (55). Additionally, childhood abuse is a very important risk factor for the development of psychopathology (56–59). The severity of the effect appears to depend on the timing of the abuse exposure (30, 60), and emotional abuse in early childhood, more than in other periods, is associated with an increase in the incidence of psychiatric diseases in adulthood and childhood (61).

Because resilience is defined as the ability to avoid the negative psychological, biological, and social consequences of stress that impair psychological and physical homeostasis, resilience is highly correlated across the spectrum of psychiatric diseases (12). The RAS, which estimates positive self-appraisals, is divided into emotion coping, situation coping, and social support subscales. In our study, the lower emotion coping of resilience, the greater the tendency the subject believed in cue–outcome associative probability in a volatile environment (increased ω_2_) in the task using social stimuli. The increased tendency to believe in cue–outcome associative probability in a volatile environment means that the degree of belief updating in each trial is high under uncertain conditions. The subjects with low emotion coping of resilience tend to fail to constantly learn about uncertain environments because the volatile environment is sufficiently stressful for the subjects. However, further studies are needed to determine whether a similar relationship exists in a ProAL task using a nonsocial association. We cautiously assume that the increased ω_2_, indicating discomposure about coping with a stressful environment, is thought to be an intermediate factor in the process of the negative emotional experience of early childhood affecting emotion coping in resilience (**Figure 1**). Namely, increased ω_2_ may be interpreted as a quantification of the individual trait that occurs during the process of early verbal abuse experiences affecting emotion coping in normal young adults.

Among the subscales of RAS, RAS-emotion coping reflects the mechanism regulating the effects of stress by cognitively controlling the emotional impact on the stressful environment. In addition, RAS-social support refers to solving difficult situational problems and RAS-social support defines the ability to obtain social support. In our study, exposure to verbal abuse and ω_2_ only explained the RAS-emotion coping score, and the scores for the remaining RAS subscales were not explained by the regression model. One potential explanation is that ω_2_, which means that model updating is affected in uncertain situations, would more likely be associated with an implied sense of emotion coping, which is the meaning of the cognitive control of emotional effects according to stressful situations, than the other two subscales of RAS.

In our mixed graphical model, “past” verbal abuse experienced in early childhood and “present” HGF learning parameters were not directly related to STAI-T (anxiety trait) but were related to RAS-emotion coping. Therefore, we are unable to eliminate the past experience, but we may reduce anxiety by improving the appraisal ability. As stress or perceived stress is potentially associated with resilience and task parameters, they should be evaluated in future studies. In addition, the HGF parameter can be used as an objective indicator of the effect of this process. The effects of the HGF parameter we suggested as a mediator requires further clinical and prospective studies.

The limitations of our study are listed below. First, this study was a cross-sectional study and was limited by the requirement to obtain recall information about verbal abuse through questionnaires. Second, although anxiety was excluded as a predictor in the regression analysis, the sample size was small compared to the variables. The mixed graphical model was used for the analysis to address this limitation. An increased sample size and cohort studies are needed. Third, the VAQ did not allow participants to reflect on the perpetrators of verbal abuse, such as peers and parents. Information about the period and perpetrators of verbal abuse would be useful, but the reliability of VAQ may decrease due to the increased number of questions. Fourth, in the case of the HGF model, the analysis of the κ free model was excluded, and κ was obtained by fixing κ. Fifth, we were unable to determine whether ω is a characteristic of the subject or a parameter that changes depending on the task. Therefore, the most appropriate interpretation of ω is a characteristic parameter of the subject when he/she is performing a specific task. Sixth, we recruited subjects from a population of young people at a single university. However, the recruitment of young adults was the best way to reduce the confounding factors due to the temporal discount. Nonetheless, we should be careful to generalize our results to all ages.

The implication of our study is that the self-appraisal of individual emotion coping, a component of resilience, was explained by “the past” (verbal abuse history) and “the present” (parameters that were obtained with an objective task). Importantly, a certain period of past negative experiences had an impact on emotion coping and ω_2_ was the individually varying trait mediating the effect on behavior on coping stress of individuals who have experienced verbal abuse.

## Author Contributions

TY and BJ designed the study. TY collected the data. DS and BJ performed the statistical analysis. and DS, TY, and BJ wrote the first draft of the manuscript.

## Funding

This research was supported by the Brain Research Program through the National Research Foundation of Korea (NRF) funded by the Ministry of Science & ICT (NRF—2016M3C7A1914448 and NRF—2017M3C7A1031331).

## Conflict of Interest Statement

The authors have neither financial nor nonfinancial competing interests to declare.
