# Supplementary material for: The Associations of Emotion Coping Appraisal With Both the Cue–Outcome Contingency and Perceived Verbal Abuse Exposure
Source: Front Psychiatry. 2019 Apr 16;10:250. doi: 10.3389/fpsyt.2019.00250 (PMC6477065; doi:10.3389/fpsyt.2019.00250)
Supplement: Supplementary file 1 [file Table_1.docx]

***Supplementary Material***

**Title: The Associations of Emotion Coping Appraisal with both the Cue-Outcome Contingency and Perceived Verbal Abuse Exposure**

Dong Woo Shin, Taekeun Yoon, and Bumseok, Jeong*

*** Correspondence: Bumseok Jeong**: bs.jeong@kaist.ac.kr

# Supplementary Figures and Tables

## Supplementary Figures


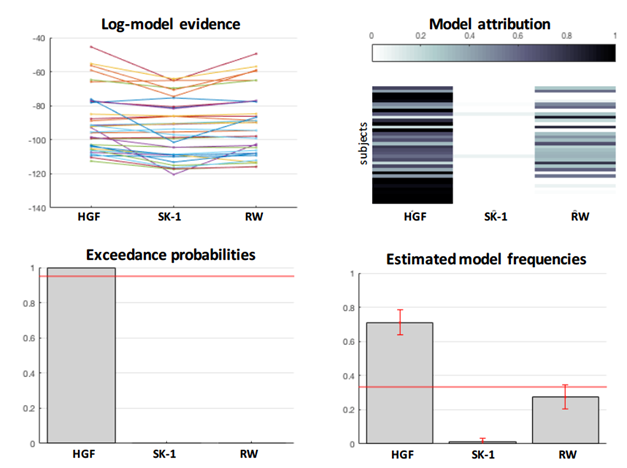


**Supplementary Figure 1.** The results of RFX-BMS. We compared four models (HGF, SK-1, and RW) in RFX-BMS. RFX-BMS yields an EP of 0.9952 and a PEP of 0.9785 for HGF, indicating that the HGF model was the best model for this task.

RFX-BMS: random-effects model comparison, HGF: hierarchical Gaussian filter, SK-1: Sutton K1, RW: Rescorla-Wagner, EP: exceedance probability, PEP: protected exceedance probability.


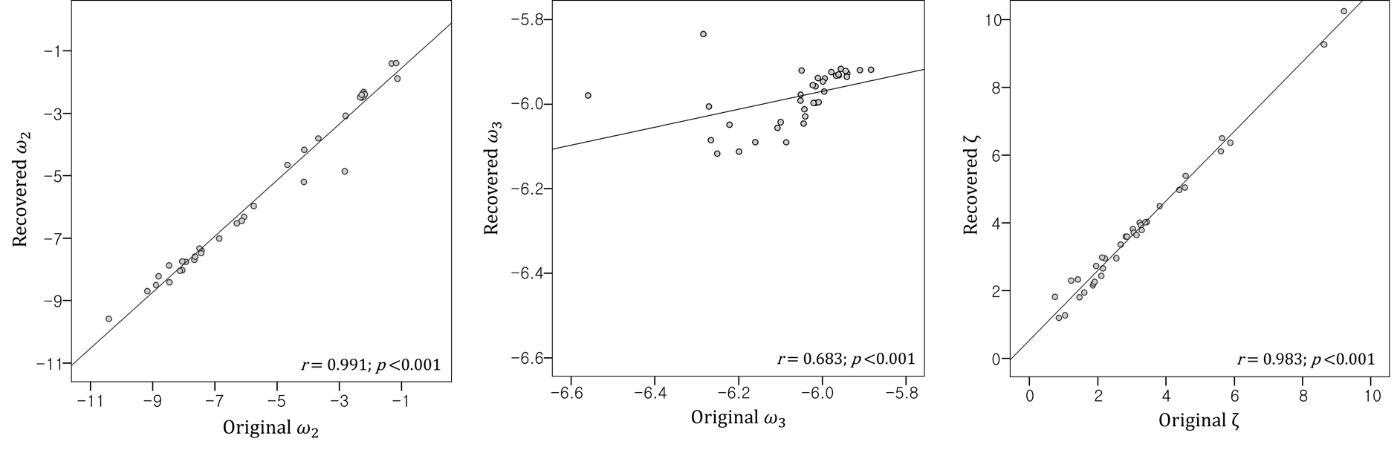


**Supplementary Figure 2.** Parameter recovery. A parameter recovery simulation was performed by simulating 100 responses using estimated parameters from each subject and subsequently re-estimating the parameters from these simulated responses. We evaluated the relationship between the original parameters and the mean of the recovered parameters 100 times. The original parameter is shown on the x-axis and the recovered parameter is shown on the y-axis. Parameters of the original model were significantly correlated with the mean of recovered parameters (Spearman’s correlation analysis), indicating that the parameters were reliably estimated.
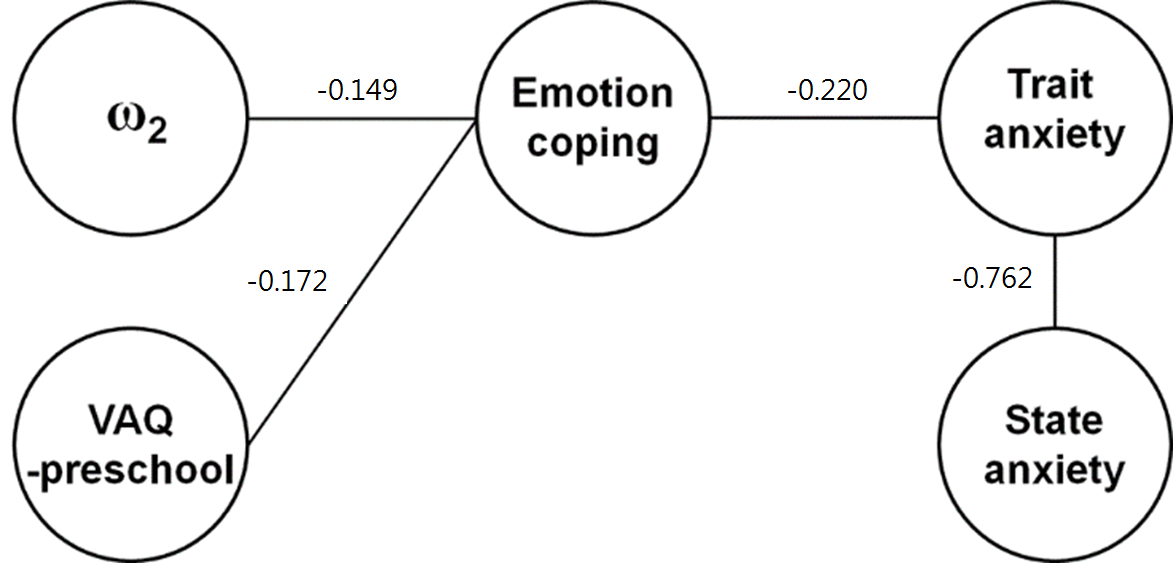


**Supplementary Figure 3.** Mixed graphical model of RAS-emotion coping with propensity score matching. We extracted data from 13 men and 13 women with propensity score matching of age and IQ to clarify whether a gender effect exists. We tested the mixed graphical model with the gender-matched subgroup consisting of 26 subjects. The mixed graphical model described the correlations of the RAS-emotion coping score with ω_2_ and VAQ-preschool, and with trait anxiety. These results are consistent with the data shown in Figure 4 in our main manuscript. The gender factor was removed from the figure because it was not associated with any parameters. The estimates using the mixed graphical model function are displayed above the connecting line.

## Supplementary Tables

**Supplementary Table 1.** Mean and variance of Gaussian priors used in HGF parameter estimation.

| HGF parameter | Prior mean | Prior variance |
| --- | --- | --- |
| Tonic volatility (level 2: ω_2_) | -3 | 16 |
| Tonic volatility (level 3: ω_3_) | -6 | 16 |
| Decision noise (ζ) | log_e_48 (= 3.8712) | 1 |

HGF: hierarchical Gaussian filter

**Supplementary Table 2.** VIF value derived from multicollinearity test for proposed models using the RAS-total score as the dependent variable.

| Predictor  Regression model | VAQ-preschool (T) | ω_2_ | VAQ-preschool (T) × ω_2_ |
| --- | --- | --- | --- |
| Simple model | 1.00 | 1.00 | - |
| Full model | 9.24 | 3.88 | 11.61 |
| Partial model with ω_2_ | - | 1.26 | 1.26 |
| Partial model with VAQ-preschool | 3.00 | - | 3.00 |

VIF: variance inflation factor, RAS: Resilience Appraisal Scale, VAQ: Verbal Abuse Questionnaire, (T): Tukey’s ladder of powers transformation.

VAQ-preschool (T) × ω_2_: interaction of VAQ-preschool (T) and ω_2_

**Supplementary Table 3.** The results of random-effects model comparison (RFX-BMS).

| Model  Estimate | HGF | SK-1 | RW |
| --- | --- | --- | --- |
| Estimated model frequencies | 0.7104 | 0.0151 | 0.2745 |
| Exceedance probabilities | 0.9952 | 0 | 0.0048 |
| Protected exceedance probabilities | 0.9785 | 0.0084 | 0.0131 |

HGF: hierarchical Gaussian filter, SK-1: Sutton K1, RW: Rescorla-Wagner.

**Supplementary Table 4.** Model fit and LOOCV result of the multiple linear regression analysis of ‘Partial model with ω_2_’, ‘VAQ-preschool only model’ and ‘ω_2_ only model’.

|  | Linear model fit | | | | | Predicted value | | | Model comparison | |
| --- | --- | --- | --- | --- | --- | --- | --- | --- | --- | --- |
| Regression model | Adjusted R^2^ | RMSE | AIC | |  | Q^2^ | | RMSE | (ANOVA) | |
| RAS-total (dependent variable) | |  |  |  | | |  | |  | |
| Partial model with ω_2_ | 0.170 | 5.091 | 227.3 | |  | 0.134 | | 5.587 |  | |
| VAQ-preschool only model | 0.101 | 5.379 | 229.3 | |  | 0.093 | | 5.719 | F(1,33) = 3.847, *p*-value = 0.0583 | |
| ω_2_ only model | 0.063 | 5.492 | 230.8 | |  | 0.029 | | 5.916 | F(1,33) = 5.405, *p*-value = 0.0263 | |
| RAS-emotion coping (dependent variable) | | |  |  | | |  | |  |  |
| Partial model with ω_2_ | 0.168 | 2.204 | 167.1 | |  | 0.142 | | 2.405 |  |  |
| VAQ-preschool only model | 0.105 | 2.320 | 168.8 | |  | 0.103 | | 2.458 | F(1,33) = 3.567, *p*-value = 0.0677 | |
| ω_2_ only model | 0.085 | 2.346 | 169.6 | |  | 0.054 | | 2.524 | F(1,33) = 4.382, *p*-value = 0.0441 | |

LOOCV: leave-one-out cross-validation, VAQ: Verbal Abuse Questionnaire, RAS: Resilience Appraisal Scale, adjusted R^2^: adjusted squared correlation coefficient, RMSE: root mean square error, AIC: Akaike information criterion, Q^2^: predictive squared correlation coefficient, ANOVA: analysis of variance.

Partial model with ω_2_: RAS ~ VAQ-preschool (T) × ω_2_ + ω_2_

VAQ-preschool only model: RAS ~ VAQ-preschool (T)

ω_2_ only model: RAS ~ ω_2_

(T): Tukey’s ladder of powers transformation

VAQ-preschool (T) × ω_2_: interaction of VAQ-preschool (T) and ω

**Supplementary Table 5.** Multiple linear regression analysis of the ‘Partial model with ω_2_’ with the dependent variable for each RAS subscale

|  | RAS Subscale (dependent variable) | | |
| --- | --- | --- | --- |
|  | RAS-emotion coping | RAS-social support | RAS-situation coping |
| ω_2_ (estimate, 95% CI) | -0.445 (-0.748, -0.142)^**^ | -0.232 (-0.497, 0.034) | -0.319 (-0.580, -0.057)^*^ |
| VAQ-preschool (T) × ω_2_ (estimate, 95% CI) | 0.119 (0.008, 0.231)^*^ | 0.101 (0.003, 0.199) | 0.086 (-0.010, 0.182) |
| Constant (estimate, 95% CI) | 13.851 (12.195, 15.507)^***^ | 16.498 (15.048, 17.948)^***^ | 15.223 (13.797, 16.649)^***^ |
| Adjusted R^2^ | 0.168 | 0.076 | 0.109 |
| Residual standard error (df = 33) | 2.302 | 2.015 | 1.982 |
| F (2,33), *p*-value | 4.528^*^ (0.0183) | 2.445 (0.1023) | 3.135 (0.0567) |

RAS: Resilience Appraisal Scale, VAQ: Verbal Abuse Questionnaire, CI: confidence interval, (T): Tukey’s ladder of powers transformation, adjusted R^2^: adjusted squared correlation coefficient.

VAQ-preschool (T) × ω_2_: interaction of VAQ-preschool (T) and ω_2_

^*^*p* < 0.05, ^**^*p* < 0.01, and ^***^*p* < 0.001

**Supplementary** Table 6. LOOCV result for each model.

| Regression model | Q^2^ | RMSE |
| --- | --- | --- |
| *RAS-total (dependent variable*) |  |  |
| Simple model | 0.117 | 5.640 |
| Partial model with VAQ-preschool | 0.065 | 5.805 |
| Partial model with ω_2_ | 0.134 | 5.587 |
| *RAS-emotion coping (dependent variable*) |  |  |
| Simple model | 0.156 | 2.385 |
| Partial model with VAQ-preschool | 0.125 | 2.429 |
| Partial model with ω_2_ | 0.142 | 2.405 |

LOOCV: leave-one-out cross-validation, Q^2^: predictive squared correlation coefficient, RMSE: root mean square error, RAS: Resilience Appraisal Scale, VAQ: Verbal Abuse Questionnaire.

Simple model: RAS-emotion coping ~ VAQ-preschool (T) + ω_2_

Partial model with ω_2_: RAS-emotion coping ~ ω_2_ + VAQ-preschool (T) × ω_2_

Partial model with VAQ-preschool: RAS-emotion coping ~ VAQ-preschool (T) + VAQ-preschool (T) × ω_2_

VAQ-preschool (T) × ω_2_: interaction of VAQ-preschool (T) and ω_2_

(T): Tukey’s ladder of powers transformation

**Supplementary Table 7.** Various multiple linear regression models using RAS-total as the dependent variable

|  | *RAS-total (dependent variable)* | | | |
| --- | --- | --- | --- | --- |
|  | Model 1 | Model 2 | Model 3 | Partial model with ω_2_ |
| VAQ-preschool (T) (estimate, 95% CI) | -1.411 (-4.146, 1.324) |  |  |  |
| VAQ-childhood  (estimate, 95% CI) | 0.033 (-0.261, 0.327) |  |  |  |
| VAQ-adolescence  (estimate, 95% CI) | -0.116 (-0.444, 0.212) |  |  |  |
| ω_2_ (estimate, 95% CI) | -0.851 (-1.849, 0.147) | -1.268 (-2.277, -0.260)^*^ | -1.004 (-1.888, -0.121)^*^ | -0.995 (-1.696, -0.295)^**^ |
| ω_3_ (estimate, 95% CI) | -2.535 (-21.865, 16.796) | -3.115 (-22.022, 15.791) | -0.647 (-19.087, 17.794) |  |
| ζ (estimate, 95% CI) | 0.065 (-1.185, 1.314) | -0.096 (-1.259, 1.066) | 0.041 (-1.133, 1.215) |  |
| ω_2_ × VAQ-adolescence (estimate, 95% CI) |  | 0.018 (0.001, 0.036)^*^ |  |  |
| ω_2_ × VAQ-preschool (T) (estimate, 95% CI) |  |  | 0.308 (0.035, 0.581)^*^ | 0.306 (0.048, 0.564)^*^ |
| Constant (estimate, 95% CI) | 31.701 (-89.083, 152.484) | 25.127 (-93.556, 143.811) | 41.485 (-74.076, 157.046) | 45.572 (41.747, 49.397)^***^ |
| Adjusted R^2^ | 0.073 | 0.103 | 0.117 | 0.170 |
| Residual standard error | 5.620 (df = 29) | 5.526 (df = 31) | 5.485 (df = 31) | 5.317 (df = 33) |
| F value, *p*-value | 1.458 (df = 6; 29), 0.2273 | 2.010 (df = 4; 31), 0.1176 | 2.157 (df = 4; 31), 0.0972 | 4.589^*^ (df = 2; 33), 0.0174 |

RAS: Resilience Appraisal Scale, VAQ: Verbal Abuse Questionnaire, (T): Tukey’s ladder of powers transformation, CI: confidence interval, adjusted R^2^: adjusted squared correlation coefficient.

Model 1: RAS ~ VAQ-preschool (T) + VAQ-childhood + VAQ-adolescence + ω_2_ (T) + ω_3_ + ζ

Model 2: RAS ~ ω_2_ + ω_3_ + ζ + VAQ-adolescence × ω_2_

Model 3: RAS ~ ω_2_ + ω_3_ + ζ + VAQ-preschool (T) × ω_2_

Partial model with ω_2_: RAS ~ VAQ-preschool (T) × ω_2_ + ω_2_

ω_2_ × VAQ-adolescence_:_ interaction of ω_2_ and VAQ-preschool

ω_2_ × VAQ-preschool (T)_:_ interaction of ω_2_ and VAQ-preschool (T)

^*^*p* < 0.05, ^**^*p* < 0.01, and ^***^*p* < 0.001
